# Role of rehabilitation amidst the COVID-19 pandemic: a review

**DOI:** 10.1186/s12967-021-03048-1

**Published:** 2021-09-04

**Authors:** Chaoran Yu, Ernest Johann Helwig

**Affiliations:** 1 Department of General Surgery, Shanghai Ninth People’s Hospital, Shanghai Jiao Tong University School of Medicine, Shanghai, People’s Republic of China; 2 Tongji Medical College of Huazhong University of Science and Technology, Wuhan, People’s Republic of China

**Keywords:** Rehabilitation, Inpatients, Outpatients, COVID-19

## Abstract

COVID-19 remains globally a highly infectious disease targeting multiple organs. Rehabilitation is increasingly valued among the supportive care fields to combat COVID-19 as currently definitive curative treatment remains largely absent. This narrative review is to address rehabilitation related topics associated with the treatment of COVID-19 patients. Nosocomial spread remains a high risk for healthcare workers, with comparable high ratios of exposed workers suffering from the disease with more severe clinical course. Primary principle of rehabilitation is to protect rehabilitation physicians and cover all person-to-person interactions. Translating perspectives are encouraged through each multidisciplinary approach. Rehabilitation for the outpatient remains a potential beneficial approach. Artificial intelligence can potentially provide aid and possible answers to important problems that may emerge involving COVID-19. The real value of rehabilitation in COVID-19 may be very impactful and beneficial for patient’s physical and mental health.

## Introduction

Rehabilitation is increasingly valued among the supportive cares to combat COVID-19 as currently definitively curative treatment remains absent [1]. Rehabilitation physicians serve effectively in each medical team for the fight against COVID-19. Early clinical recommendations to translate rehabilitation into the explorative treatment of patients with COVID-19 were mainly extrapolated from the studies of patients with prior SARS/MERS epidemic or other pulmonary diseases [1, 2]. However, more unique rehabilitation challenges are arising from COVID-19 as the pandemic is robustly growing [1].

The rehabilitation of pulmonary function in COVID-19 patients covers several tailored therapies adapted from the American Thoracic Society/European Respiratory [1, 3]. The therapies include exercise training, education and relevant clinical interventions that aim to reduce complications, minimize disability and secure sufficient physiological functions and life quality [1, 4].

COVID-19 remains a highly infectious disease targeting multiple organs without specific treatment except for the possibility of prevention with the forthcoming vaccinations [5]. By far, the severity pattern of COVID-19 can be categorized into four levels: asymptomatic infection cases, symptomatic cases isolating at home, symptomatic cases admitted to hospital, and severe cases requiring medical interventions like ventilation [5]. Rehabilitation services stand out among full multidisciplinary treatments when COVID-19 pressures the routine medical services with significant morbidity. The National Institute for Health and Care Excellence suggests early initiation (first 30 days, post-acute phase) of progressive rehabilitation programs exert the most benefits on recovery [6]. In fact, rehabilitation medicine serves as the key service for the sequelae of those surviving COVID-19 with a comparable high cost of time and resources [5, 6]. Years of rehabilitation practice is recommended to be at the forefront of COVID-19 patients at chronic phase [6]. Evidence-based consensus has provided direct guidelines and recommendations of rehabilitation for COVID-19 patients [6]. However, these recommendations require further evidence and field experience to refine and upgrade. Great efforts have been made in defining a framework of rehabilitation for COVID-19 patients with an evidence-based approach, such as employment of a multidisciplinary expert team and four evidence-based classes of interventions (exercise, practice, psychological support, and education particularly about self-management) [7].

On 17, January 2020, Dr. Leopold wrote an editorial, a conversation with Dr. Parekh, addressing the waste and fraud in the United States healthcare system [8]. Given the huge amount of increasing healthcare spending from 2005 to 2015, worsening administrative bloat of healthcare system was detected, instead of expected high efficiencies [8]. Of note, waste in healthcare system, including failure of care delivery or coordination, administrative complexity and other issues, accounted for around one-quarter of all healthcare cost in the United States, about 1 trillion USD [8]. Substantial waste regarding duplication and redundancy is noticed in care management, quality improvement and population management [8]. To bend the waste curve, Dr. Parekh suggested using technology to streamline burdensome processes, such as Centers for Medicare & Medicaid Services’ (CMS) Blue Button 2.0, or systems to change practice patterns, such as High-value Healthcare Collaborative [8]. Also, evidence-based practices, as Dr. Parekh highlighted, are one of the high-valued interventions for patients, but difficult to disseminate [8].

Serving as a constitutive part, the rehabilitation discipline’s contribution in the fight against COVID-19 is significantly growing, and is likely to be a breakingpoint for waste control and dissemination of evidence-based practice in US healthcare system.

During the pandemic periods, the healthcare system is under tremendous pressures to live-saving task and costs. The present and future role of rehabilitation medicine will not be the role it was in the past. How the wastefulness curve changes and how rehabilitation medicine reacts to this pandemic remain unclear. Since CMS provides effective administration over evidence-based practice, it is still debatable how to decide which is evidence-based rehabilitative practice and which is not [8]. Commonly, prior to COVID-19, literature review was performed in specific areas to educate what were the best practices by CMS. During the pandemic, a huge amount of literature has been on display. This is feasible for short term rehabilitation since most resources have been focused on COVID-19 patients with acute and subacute phase. For those entering chronic phase, the evidence of rehabilitation remain comparably weak or still mounting. Noteworthy, long- COVID, also known as COVID-19 long-haul syndrome, or post-acute COVID-19 syndrome, defined by prolonged infection-related symptoms, such as fatigue, respiratory complaints and cognitive impairment, accounts for up to 10% of total COVID-19 patients [9]. These patients are not severe enough to hospitalization, but a prolonged negative impact remains on their daily life [10]. It is reasonable to presume that long term rehabilitation practices will be the next intensively studied issue, with global rehabilitation initiative commissioned by World Health Organization (WHO) and related rehabilitation education tools for professional.

This narrative review is to address rehabilitation related topics associated with the treatment of COVID-19 patients.

## General principles of rehabilitations for COVID-19

The primary principle is to protect rehabilitation physicians using protective equipment such as gloves, masks, or isolation gowns to cover all person-to-person interactions [11, 12]. Nosocomial spread remains a high risk for healthcare workers, with comparably high ratios of exposed workers suffering from the disease with more severe clinical course [1, 11]. Viral shedding from asymptomatic COVID-19 patients significantly contributes to the nosocomial infections [11]. Reduction of unnecessary interpersonal contact can be a key to prevent widespread disease distribution.

Self-monitoring patterns are strongly encouraged during this pandemic. Rehabilitation should be performed in a self-supervised manner via telemedicine if possible. Previous studies have reported that equal outcomes were achieved between telehealth rehabilitation programs and center-based programs [13]. Moreover, internet-based rehabilitation programs also improve both painless life quality and physical activity [14–16].

## Rehabilitation for inpatient

Patients diagnosed with respiratory distress or symptomatic discomforts, combined with abnormal oxygen saturation meet the hospitalization criteria [13]. Respiratory rate > 30 times each minute, oxygen saturation < 93% and PaO2/FiO2 < 300 mmHg are the most frequently used findings for monitoring [1, 13].

**Fig. 1:**
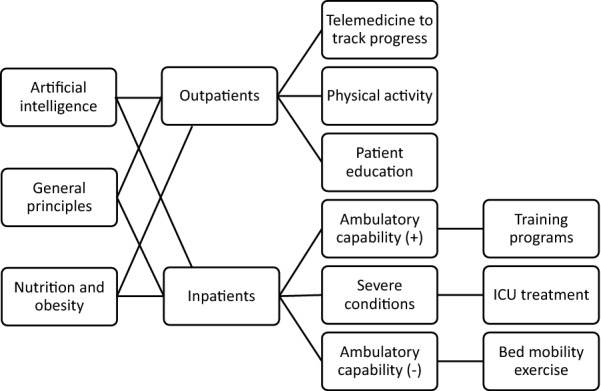
Flowchart of clinical management for patients with COVID-19

The work principle for the hospitalization of COVID-19 patients is clear (Fig. 1). Once admitted, systemic assessment is required. Patients with severe condition are sent to intensive care unit (ICU) for both medical and rehabilitative treatment. Patients without ambulatory capability will receive bed mobility exercise and muscle training. Patients with ambulation will be under frequent assessment combined with progressive training programs [1].

Interestingly, 4S principle (simple, safe, satisfy and save) of pulmonary rehabilitation guidelines for COVID-19 patients have been established in China, in order to build a practical and feasible rehabilitation method in response to the extraordinary pressure [17]. This 4S principle is both applicable to inpatient and outpatient management.

### Role of rehabilitation in ICU

Although early rehabilitation is beneficial to general COVID-19 patients, the exact performance of rehabilitation in ICU may not be the same as it is for inpatients outside ICU. High risk of nosocomial spread in ICU remains a major consideration. Procedures such as tracheal intubation and mechanical ventilation in ICU considerably enhance the exposure of bioaerosol, exerting a hostile environment. Solid evidence regarding the exact role of rehabilitation in ICU remains vacant. However, muscle training is essential to patients with high risk of respiratory muscle weakness due to prolonged mechanical ventilation [1].

### Modified Borg Dyspnea Scale (MBDS) in rehabilitation

MBDS is commonly used to measure dyspnea in patients with chronic lung disease such as pulmonary arterial hypertension (PAH) [18, 19]. A 0 to 10 numerical score is given to assess dyspnea status of the patient receiving 6-min walk testing [19–21] (Table 1). MBDS has been identified as an univariate mortality predictor in patients with PAH [22]. During the physical exercise of rehabilitation, MBDS is an essential predictor to manage the process [1]. If the score > 3 combined with unstable SpO2 and oxygen supplementation, physical exercise is recommended to pause [1, 23]. In addition if chest pain, palpations and dizziness occur, the activity is also paused [1]. MBDS is a reliable tool both for rehabilitation physicians to make assessment and patients to self-monitor.

**Table 1:** Modified Borg Dyspnea Scale

Score	Description of breathlessness
0	Not at all
0.5	Very, very slight (just noticeable)
1	Very slight
2	Slight
3	Moderate
4	Somewhat severe
5	Severe
6	
7	Very severe
8	
9	Very, very severe (almost maximal)
10	Maximal

### Exclusion criteria of inpatients rehabilitation

Rehabilitation does not apply to inpatient when severe or critical conditions occur, such as loss of consciousness and acute myocardial ischemia [1]. Moreover, rehabilitation is also recommended to exempt patients with progressive symptoms such as rising temperature, acute dyspnea, SpO2 below 90%, abnormal blood pressure either over 180 mmHg or below 90 mmHg, abnormal respiratory rate or heart rate, any symptom of shock or systemic inflammatory response syndrome [17, 24]. In fact, around 5% of apparently healthy patients could progress into severe conditions [23].

### Translating muscle-up tutorial into physical exercise of rehabilitation

Original bulking up training in the past was strictly seen in bodybuilders. Garber and Krouskop discovered the effects of body building on the pressure distribution in patients with physical disability [25]. Some bodyweight movements without equipment that were beneficial to body coordination and strength, are also promising for the physical exercise of rehabilitation, for example, crunches, push-ups, squats and walking lunges. Design tailored physical exercise tutorials inspired by bulk up may be easier for inpatients to accept and independently perform. Here to briefly describe the crunches movement: 1. Lie on bed with both legs flat on the level. 2. Arms crossed in front of chest. 3. Go up as lifting shoulders upwards slowly using abdominal muscles and slowly back down. 4. Repeat two to three sets of 8–10 as see fit. Meanwhile, stretching, such as upper chest and lateral chest stretching, is also beneficial to pulmonary rehabilitation by increasing compliance as much as 50 ml [1].

### Combinational utilization of breathing and airway clearance techniques

All involved techniques include autogenic drainage, airway clearance, air stacking, glossopharyngeal breathing, forced expiration maneuvers and targeted positioning. Application of these techniques enables secretions collections and airway clearance, resulting in recruitment of lung volume [1]. All referred techniques should be used based on expertise. In fact, physical exercise serves as an effective contributor to airway clearance [12, 26]. However, simple mucus airway clearance from the peripheral to upper airway may not be as effective as physical exercise [24]. Both air stacking and glossopharyngeal breathing provide extra tidal volume to maintain respiratory function [27, 28]. Another important technique is positioning. It is easier, simpler and preferable to other techniques during the rehabilitation of COVID-19 inpatients. Patients could choose sitting or standing position to facilitate lung volume, or in critical condition, choose targeted positioning. Dentice et al. reported that side lying duration of 2 min provide a sufficient ventilation/perfusion result [29]. Position duration longer than 3 min significantly worsened dose disparity [29]. To enhance ventilation, all positions are recommended, including side lying, upright, supine, sitting and others.

### Obesity and COVID-19

Obesity continues to be a significant risk variable for COVID-19 patients, particularly in the ICU [30]. More severe courses of COVID-19 infection are found in people with excessive BMI. Overweight BMI is found in over two thirds of ICU patients [30, 31]. Similar results were also reported during influenza A pandemic in 2009 [31]. How exactly obesity affects the disease course is yet to studied. Until now, human angiotensin-converting enzyme-2 (ACE-2), a protein highly expressed in pulmonary alveolar epithelial cell and endothelium, has shown high affinity to the receptor of COVID-19 [32]. Commonly, researchers believe ACE-2 is the entry of COVID-19 into human body through activating the signal pathway of renin-angiotensin-system (RAS) [33]. Among all types of tissues, adipose tissue may show higher affinity to SARS-CoV-2 than lung tissue due to higher expression level of ACE-2. However, direct evidence to support this conclusion remains absent. Patients taking the ACE inhibitors or angiotensin receptor blockers for hypertension treatment are suggested to keep using their prescribed medications [34, 35]. Meanwhile, some proinflammatory adipokines and cytokines that link the immune reaction and the severity of COVID-19 are generated by the adipose tissue [36]. However, no specific obesity guideline has been published targeting the treatment of COVID-19 patients with obesity. Weight loss by hypocaloric diet and physical exercise should be further considered in rehabilitation therapeutic guideline.

### Nutrition intake in the course of COVID-19

Nutrition intake is important for the normal immune function. Older COVID-19 patients are prone to develop polymorbidity, the presence of at least two chronic diseases, with aging-associated factors such as oral and chew problems, cognitive impairment. The European Society for Clinical Nutrition and Metabolism (ESPEN) proposes that COVID-19 patients with malnutrition show higher mortality and worst clinical outcomes [37]. Nutrition optimization guidelines from ESPEN have been released [37]. Briefly, energy, protein and fat carbohydrate needs are specified based on each individual scenario (Table 2). Micronutrient insufficiency is also commonly noticed during the course with significant risk of worse outcome in COVID-19 patients. Between 9 December, 2019 and 13 December 2020, case fatality rates significantly increase in severe-selenium-deficiency areas (3.16%) comparing to non-selenium-deficiency areas (1.17%) [38]. The role of fruits and vegetables consumption has been recognized to make an impact on immune system [39]. Thus, vitamins family, folate, zinc, copper, selenium, iron and unsaturated fatty acids should be added in daily nutrition intake.

**Table 2:** Nutrition intake parameters based on The European Society for Clinical Nutrition and Metabolism (ESPEN) guidelines

	Patient type	Recommendation
Energy needs	Polymorbid patients aged > 65 years	27 kcal per kg per day
	Severely underweight polymorbid patients	30 kcal per kg per day
	Older persons based on individually comprehensive adjustment	30 kcal per kg per day
Protein needs	Older persons based on individually comprehensive adjustment	1 g protein per kg per day
	Polymorbid medical inpatients	≥ 1 g protein per kg per day
Fat: carbohydrates ratio	Patients without respiratory deficiency	30:70
	Patients by ventilation	50:50

## Rehabilitation for outpatient

Even though the need for standardization of inpatient treatment remains urgent, the most common form of treatment and rehabilitation remains the outpatient approach. On December 3rd, 2020, California saw 200,836 COVID-19 tests done, had confirmed 22,018 new positive tests, and had 2152 ICU positive patients [40]. This, on a random day, shows that most patients who have a COVID positive do not necessarily progress to the inpatient level but will most likely remain at home for quarantining and rehabilitation. Isolating at home is an important step to prevent spread, which in addition to precautions like handwashing and facemasks, even at home, can help avert dissemination amongst isolated people. Luckily, through the aid of technology, services like telemedicine are available to help track progress remotely and prevent further unnecessary spread.

Struyf et al. reviewed studies of signs and symptoms that were commonly used to determine if patients had COVID and found that there were over 25+ symptoms possibly related to COVID [41]. Even though they do not appear together as a constellation, having this number of potential symptoms to treat is not a desirable position for physicians or patients as it means there must be varying degrees and approaches to treatment, mostly symptom specific. Being a respiratory disease, certain predictable symptoms like fever, cough, productive sputum, sore throat, shortness of breath, and others can appear, though the commonly reported symptom of anosmia or ageusia have perplexed researchers and medical experts.

Initial proposals for mild management by Wang et al. highlight the need for physical activity, breathing exercises and airway clearance [1]. Recommendations for physical activity include light exercise with a score of ≤ 3 on the Borg Dyspnea Scale for intensity at a frequency of 1 or 2 times per day, 3 to 4 times a week. The recommended duration varies depending on specific activity and starts at roughly 10 to 15 min for the first sessions and is gradually increased to 15 to 45 min with increase in workload and effort in subsequent sessions. Recommended activities include walking and biking (if conditions permit and social distancing protocols are followed). Breathing exercises are encouraged and involve various types of focused breathing techniques. These include diaphragmatic breathing, abdominal contractions, yoga, singing and others. Airway clearing via expectorants is helpful but should be done appropriately and with a focus to try and prevent the spread of particles. Additionally, the Huff cough technique is a less strenuous way to help clear airways without overexertion. Another important facet of their suggestions includes general patient education focusing on lifestyle modifications and psychological counsel or resources. Supplementally, the standard treatment plan of consuming appropriate fluids and simple pharmaceutical interventions such as antipyretics and analgesics function as additional helpful aids [42].

In most cases the avenue of conservative therapy will suffice, though there are certain population groups that may require extra attention. Patients suffering from hypertension, diabetes or certain types of cardiovascular issues are groups that have been found to higher admission rates than the general public. The Centers for Disease Control and Prevention (CDC-USA) found that patients with severe diabetes had a higher prevalence of severe COVID-19 [43]. Furthermore, Singh et al. analyzed 6 various studies comparing ICU care and found that all 6 studies showed an increased severity of COVID-19 in diabetics [44]. Theories as to why diabetes patients require extra concern deal with diabetes pathophysiology or modifications made by the virus to the body. Singh et al. note that SARS CoV-1 and SARS CoV-2 both utilize the ACE-2 receptors for gaining entrance into the cell leading to an assortment of changes in the body including increased ACE-2, increased furin, and altering Il-6 [44]. Changes also lead to an overall modification of innate immunity with various cell lineages being affected including NK cells and myeloid cells, even extending to adaptive immunity and T cells [45]. For diabetic patients, special attention is especially focused on controlling blood glucose level adequately, general diabetes management and potential effects from altering or changing medications, this due to the frequency of patients with diabetes already being on ACE/ARB medication. The general precautions and strategy for outpatient rehabilitation should be followed with certain modifications. An emphasis on balanced dieting for glycemic control, supervising insulin and antidiabetic drugs, and consideration of their feet and related problems should be stressed to outpatient individuals with diabetes [44]. Real challenges of preventing outpatient cases from becoming inpatient care may be due to increased amount of viral load diabetics may deal with, alveolar dysfunction, endothelial dysfunction that can also lead to issues with coagulopathy [45].

For hypertensive patients and patients with heart conditions, interaction with angiotensin II receptors can provide certain challenges since the virus uses ACE-2 receptors as an entry point that can potentially lead to alterations at the physiological level or changes in necessary medications. Kulkarni et al. point to the connection of HTN and left ventricular hypertrophy and fibrosis as reason to which patients may be at higher risk and further point to findings in other studies that indicated COVID-19 patients show higher than normal myocardial damage through cardiac biomarkers like troponin I and lactate dehydrogenase (LDH) [46]. Hypertension is thought to carry roughly a 2.5 times higher risk of developing a more severe COVID-19 infection or risk of dying, especially in elderly populations [47]. As in the diabetic community, outpatient individuals require standard rehabilitation and it is recommended that they acquire home blood pressure monitors, as this allows for less strain on primary and secondary health care services during a pandemic and encourages patient interaction in their own health [46]. Any necessary titration or consultation should be done via telehealth and in-person visits should be reserved for extenuating circumstances. The main issue is whether to maintain or discontinue the use of ACE or ARB pharmacotherapy as it is unclear whether increased ACE-2 expression can have the effect of increase the susceptibility or risk of SARS-CoV-2 [46]. Since the data for RAAS and COVID association is so scarce, there is no real clear clinical evidence to point to, but confusion may stem from differentiating ACE-1 and ACE-2 in the RAAS system and mechanism of actions of medications, namely ARBs [48]. Meng et al. postulate that RAAS medications can have a positive impact on the inflammatory response leading to a decrease in IL-6 levels and benefitting the immune pool of T Cells by inhibiting their depletion, which would help at-risk patients [49].

The unique symptoms of ageusia and anosmia have managed to baffle scholars due to their unforeseen appearance in the list of common COVID-19 symptoms. The SARS-CoV-2 virus relies on the spiny S1 protein that attaches to ACE-2 receptors, which is distributed throughout the nervous system and manages to interfere with certain nerves. In the case of anosmia, the nerves effected are the olfactory bulb and olfactory nerve, which because it is a respiratory disease, sees increased viral load in that region eventually leading to inflammation and disruption [50]. This symptom may be important and even potentially indicative of disease severity as admitted patients were roughly 10 times less likely to report the symptom of anosmia [51]. There are currently no guidelines for treatment of anosmia, though there is conflicting thought on how it should be treated, as some groups like the WHO initially felt giving corticosteroids may make the situation worse by exacerbating the disease in those with non-severe COVID-19, or others who feel that corticosteroids may be beneficial to decrease inflammation in effected zones thus regaining function [51]. For ageusia, there is no current treatment available and the condition seemingly resolves itself slowly [52]. For both symptoms, the common timeframe of resolution is estimated at 14 days after clearing the virus and both gradually return to normal.

Currently, there is no specific outpatient pharmaceutical intervention approved by the Food and Drug Administration (FDA) in the United States, as most interventions are reserved for hospitalized patients. The approval of Remdesivir for admitted patients has given physicians a useful tool for combating the virus, but application for this medication has yet to be approved or had focused trials for outpatient use [53]. Even the other FDA approved alternative medication, Baricitinib, must be used in conjunction with Remdesivir in individuals already requiring some assistance breathing. Likewise, Dexamethasone is recommended only for severe COVID-19 infections, which is classified as having respiratory distress and altered respiratory rate and oxygen saturation, though, there may be special considerations for pregnant women with mild diagnosed COVID to take antenatal steroids after benefit/harm analysis is done for mother and child [54]. Future potential therapies seem aimed at components of inflammation like IL-6 and others, or therapeutic antibodies with monoclonal antibodies [55].

The significance of having dedicated outpatient strategies cannot be stressed enough, as there is a vast difference between a 2-week disease and something that is life threatening. The goal for outpatient therapies should be conservative and ideally to prevent the long-term harm, unwanted aftereffects or even for the need of admission. Disease unpredictability can be seen in various parts of the body including heart through cardiac injury, neurological issues as seen in cases where anosmia or ageusia are persistently present, interactions with pre-existing conditions or even negative psychological effects from sickness, so naturally the aim of preventing disease severity from increasing in outpatients is paramount [56].

## Incorporating artificial intelligence and deep learning to improve health outcomes

Though in its infant stage of true implementation, artificial intelligence, also referred to as deep learning or machine learning, can potentially provide aid and possible answers to important problems that may emerge involving COVID-19. Thus far, early attempts to apply machine learning or deep learning to solving the COVID puzzle have included: screening of SARS-CoV-2 assays, development of methods for screening depending on patient’s breathing patterns, and analysis of CT imaging [57]. Other important ways Vaishya et al. believe AI can be utilized is with monitoring treatment and extracting visual data for disease progression, finding levels of infections through contact tracing, projecting infection rates and mortality, studying data to create or enhance medications, and finally for general prevention and reduction of workload on healthcare systems [58]. Meanwhile, AI can be useful to make early detection of long-haul symptoms. Since significant clinical features between initial and post-acute COVID-19 syndrome have been distinguished (Table 3), similar data can be retrieved for training of AI-based algorithms, enabling to build early risk assessment models.

**Table 3:** Clinical features comparison between initial stage and post-acute COVID-19 syndrome patients

Clinical features [9]	Initial stage (% of patients)	Post-acute (% of patients)
Fever***	60	10
Shivers***	56.7	13.3
Myalgia	76.7	53.3
Arthralgia	43.3	46.7
Cough***	73.3	26.7
Dyspnea	80	46.7
Thoracic oppression	83.3	56.7
Diarrhea	50	30
Nausea	23.3	10
Anosmia*	43.3	10
Fatigue	93.3	82.8
Thoracic pain	36.7	23.3
Cephalalgia	36.7	36.7
Paresthesia****	0	60
Burning pain**	0	43.3

^*^ < 0.05, ** < 0.01, *** < 0.005, **** < 0.001

An important step in the rehabilitation process is seeing who would benefit the most from certain interventions, Pu et al. used machine learning to evaluate differences in treatment plans [59]. This type of comparative analysis helps personalize medicine to the individual and helps clarify different routes that may be available for patients, avoiding the “one size fits all” approach that may persist. Hassanien et al. employed machine learning to better understand and forecast how a patient may react to the disease and the possibilities they may have for recovery [60]. While prioritizing certain patients is not a simple excursion, there are scenarios in which this needs to be done, mostly due to resources and time being finite and in order to give the patient the best chance at recovery at the appropriate time. Prediction models that would exponentially help healthcare providers and have been postulated include likelihood of ICU visit, or use of biomarkers for mortality, which can help highlight those who are most at risk so resources like allocation of respirators or general case severity can be understood and immediately acted upon by providers [61]. Tools like these are essential for elucidating the possibilities for improved patient recovery.

In the coming years, there is no doubt that eventually there will be designated pharmaceutical treatments that will address SARS-CoV-2 infections. However, a general question that is asked is “Do we have enough time?” or “Have we studied this enough to implement broadly?”. This is the scenario currently seen by health providers doing their best to help patients, pharmaceutical companies trying to create medications, and the general public who is waiting for a tried and tested treatment but is also skeptical about a rushed finished produce and how much research it has undergone. Interestingly, this may be an area in which AI may be most helpful. Early in 2020, Stebbing et al. used BenevolentAI, an AI algorithm to see if there was a way to repurpose drugs. In this case, they used the previously mentioned Baricitinib, an immunosuppressant used in rheumatoid arthritis that can also perform the indirect functions of an antiviral to slow down infection, to see if there was any possibility it could be employed in the fight against COVID [62]. One important reason for attempting this is time, as repurposed drugs require less research and development time, and already provide a foundation of pharmacokinetics and pharmacodynamics.

BenevolentAI predicted Baricitinib could effectively be used as a substitute anti-viral since it inhibited certain essential processes that were involved in viral entry [63]. This led to experiments into Baricitinib’s anti-inflammatory against NK Cells, CD4 and CD8 T Cells, Il-6 and others, which led to the conclusion that viral load could be reduced [63]. Next, permission was given to use the medication via expanded access, or compassionate use, in 4 patients with pneumonia who subsequently saw improvements in fever and cough with decreased viral loads. This then led to a deeper randomized, placebo-controlled, double-blinded study with 1033 people enrolled and led to the discovery that Baricitinib + Remdesivir being superior to just Remdesivir alone, especially as those with high-flow oxygen or non-invasive ventilation were found to have reduced recovery time and improved clinical outcomes [64]. Scenarios like this show evidence how AI can be used to affect health outcomes in the future.

Abd-Alrazaq note that most AI studies involving COVID come from China, due to it being the origin of the disease and being one the leaders in the AI field, and that most studies to this point have lacked a focus on contact tracing, education for health care workers, or how robotics may potentially be implemented [65]. Something that cannot be understated is that a big challenge for AI’s inclusion into the general conversation comes from the fact that the disease is relatively new compared to others, which most importantly means that data pools to improve or refine algorithms are currently scarce. To ensure more precision, data of all kind, including epidemiological, clinical, diagnostic, and others, needs to be collected, which can then be used to find new patterns, unseen or overlooked concepts, and important clues that can assist physicians in finding the most optimum treatments for diseased patients.

## Perspective and prospective

There is simply no way to underestimate the impact that the COVID-19 virus has had on the world at large. Almost every single facet of daily living has some way been altered or changed in some manner. A basic chore like grocery shopping saw consumer behavior drastically change normal consumption to panic-buying and hoarding which included reasonable items like water or pasta and unreasonable products toilet paper and milk [66]. The health concerns of buying groceries during a pandemic are not just limited to being in public, hence, being potentially exposed, it can also include what products people buy and how choices trickle into unlikely things such as stay-at-home orders for COVID prevention causing further changes to overall mental and physical health of patients. This can be seen in the sales of alcohol during the pandemic. The National Institute on Alcohol Abuse and Alcoholism (USA) estimates that there was a roughly 20% increase in spirit sales for the states of Arkansas and Tennessee, and a 63% increase in the state of Delaware in May 2020, when compared to the 3 previous years averages [67]. Interestingly, certain surveys of European countries like Italy and Turkey have shown that smoking and tobacco consumption have decreased and individuals attempting smoking cessation has increased, most likely due to COVID being a respiratory disease [68, 69].

Another important consideration is the effect COVID has had on employment and people’s general mental health during the pandemic. In the United States, the U.S. Bureau of Labor Statistics found that at the start of the pandemic there was an increase in the unemployment rate, which in February 2020, showed unemployment which was at 3.8%, the lowest mark in roughly 50 years, skyrocket to roughly 14.7% in April [70]. The hardest hit groups were those in some of the most vulnerable demographics: females, minority and immigrant populations. In different parts of the world people are seeing volatility in their stock markets, increased unemployment and uncertainty in their economies and once again, the most vulnerable individuals are those in developing countries [71]. The importance of having job security and income, or lack thereof, is a source of both relief and stress to individuals. The lack of employment due to the virus, coupled with the virus itself being harmful, has led to an increase in adverse mental health challenges. A CDC survey conducted between June 24th and June 30th, 2020 found that 40.9% of respondents showed a minimum of 1 adverse behavioral or mental health condition, that included anxiety, depression, and trauma- and stressor- related disorder, and 13.3% of individuals reported they had started, or increased substance use a means to cope with the pandemic [72]. This is a troubling picture when compared to previous data from 2019 that showed a 3 × increase in anxiety related symptoms (25.5% to 8.1%) and 4 × increase in depression related symptoms (24.3% to 6.5%). Troublingly, there was a more than 2 × increase in people who had “seriously considered suicide in the past 30 days” when compared to 2018 statistics (10.7% to 4.3%) [72]. Even in things that we do not perceive as being related to COVID infection, things like shopping habits, economics or mental health, we see the virus indirectly manifest itself and exert influence the public’s general health.

While not a panacea, the development of 2 vaccines, Moderna and Pfizer-BioNTech, represent an important milestone in the pandemic and important step in an optimistic direction. The 2 vaccines, while not undergoing the longest trial periods, have been approved for emergency in the United States by the CDC and symbolize that scientists and researchers are working hard to find a solution to this overwhelming situation [73]. Ideally, the efficacy of the vaccine should inspire individuals in the larger public to seek immunization, while continuing practical prevention strategies to mitigate disease spread. The dissemination of new information is incredibly valuable and essential, though it can also be alarming as seen with the recent supposed discovery of a UK COVID variant that has led to lockdowns in the region [74]. It is important to stress to the general public that there is a universal strategy so that there is an understanding that there is a general direction and things will gradually improve if we employ a pragmatic approach to ending the pandemic. Information that would be essential to the general public should include steps being taken to combat the disease such as research or trends, addressing disease forecasts, effective means to receive healthcare and strategies that allow for people to earn a living and keep employment during the pandemic. This would hopefully reduce the direct health threat of the COVID-19 virus and help ease any mental health and indirectly health effects that might occur.

## Conclusion

At a quick glance, it is easy to think that rehabilitation for a primarily respiratory virus like SARS-CoV-2 could be straightforward or in a manner that we normally see in other respiratory illnesses, but the challenges brought on by COVID-19 pandemic extend further than that. While outpatient treatment does remain standard when compared to others in its category, the real difficult lies in the novelty of the virus and how the evolution of inpatient cases has changed during discovery and subsequent research. As more data is gathered, treatment is further optimized and improved. Questions do remain about the potential sequelae that individuals may be forced to deal with in the future as it has only been roughly 1 year since the disease continued. Another important task is providing mental health solutions to those who had been adversely affected by the pandemic, whether they be from the anxiety or depression caused by the event or loss of livelihood.

## Data Availability

The data and material supporting the conclusion of this article are included within the article.

## Abbreviations

CDC: Center for Disease Control and Prevention (USA)
FDA: Food and Drug Administration (USA)
CMS: Centers for Medicare & Medicaid Services’
ICU: Intensive care unit
MBDS: Modified Borg Dyspnea Scale
PAH: Pulmonary arterial hypertension
ACE-2: Angiotensin-converting enzyme-2
RAS: Renin-angiotensin-system
ESPEN: European Society for Clinical Nutrition and Metabolism
LDH: Lactate dehydrogenase
